# Editorial: The Clinical Neuroscience of Music: Evidence Based Approaches and Neurologic Music Therapy

**DOI:** 10.3389/fnins.2021.740329

**Published:** 2021-09-20

**Authors:** Michael H. Thaut, Gerard Francisco, Volker Hoemberg

**Affiliations:** ^1^Department of Music and Health Science, University of Toronto, Toronto, ON, Canada; ^2^University of Texas Health Science Center at Houston, Houston, TX, United States; ^3^SRH Gesundheitszentrum Bad Wimpfen GmbH, Bad Wimpfen, Germany

**Keywords:** music, neurorehabilitation, neurologic music therapy, brain imaging, innovation, brain

Modern music therapy, starting around the middle of the Twentieth century as an organized profession has traditionally been rooted in concepts of social science. The basic value of music in therapy was considered to derive from emotional and social functions in personal life experiences and a society's culture. Emotional expression, social roles to facilitate group association, integration, and relationship building, as well as general concepts of well-being and health were considered and are still today core functions to explain music as a therapeutic modality.

As the content of this special Research Topic clearly indicates, driven by new insights from research in music and brain function, a new understanding of the capabilities of music as a complex auditory language in therapy and rehabilitation has emerged over the past 25 years. Research has shown that music engages complex perceptual, cognitive, affective, speech/language, and motor control processes in the human brain. Furthermore, translational research approaches have shown that brain processes in music perception, music cognition, and music production can engage and shape non-musical perceptual, cognitive, language, and motor functions to effectively retrain the injured brain in neurorehabilitation and neurodevelopment. Music has become a language of science again as well as a new language to change the brain.

This transition is well-reflected in the research studies in this special topic as a critical step in the historical understanding of music as therapy. Rather than viewed as an ancillary or complimentary discipline to enhance other core therapies music has now been accepted to be applied effectively in core areas of training and retraining the injured or developing brain in motor, speech/language, and cognitive domains.

The most clearly developed clinical model has been encoded in Neurologic Music Therapy [NMT] which was formally established in 1999/2000. Translational biomedical research in music had led to the emergence of scientific clusters of scientific evidence for specific music based interventions. In the late 1990s researchers and clinicians in music therapy, neurology, and rehabilitation and brain sciences classified these evidence clusters into as system of standardized therapeutic techniques for sensorimotor, speech/language and cognitive rehabilitation. NMT is medically recognized as evidence based therapy [e.g., by the World Federation of Neurorehabilitation], is represented in over 60 countries by certified NMT-Therapists, and one prominent NMT technique [Rhythmic Auditory Stimulation for gait rehabilitation] has been included in the official clinical stroke care guidelines in the US [Departments of Veteran Affairs and Defense] and Canada [Heart & Stroke Foundation], Similar efforts are underway in several other countries.

This special topic represents an important step to further advance the scientific basis of this exciting development. Fourteen studies cover the breadth of new research from clinical trials to investigations of neural mechanisms underlying clinical NMT techniques to comprehensive reviews of the current evidence status of music-based interventions. The topic also includes projects breaking new ground, e.g., looking at signal coherence in EEG hyperscanning between parents and non-verbal children (Samadani et al.), including different art forms beyond music in the aging brain (Alain et al.), and NMT applications to motor deficits in neurotoxic cancer therapies (Ghai and Ghai).

It comes as no surprise that of the 14 papers half are dedicated to music in motor rehabilitation and the other to applications in the cognitive rehabilitation domain.

Included clinical research focuses on NMT in gait (Crosby et al.; Mainka et al.) and upper extremity training in stroke (Nikmaram et al.; Tian et al.). The study by Buard et al. combines clinical outcome measures with MEG based brain connectivity measures for upper extremity training in Parkinson's disease. The study by Koshimori et al. is looking at the effect of rhythmic auditory cues on dopamine release in the basal ganglia in young health adults as a prequel to further studies with Parkinson's disease. Music-based attention interventions are the topic of studies by van Alphen et al. and Kasuya-Ueba et al.. Berger Morris et al. take an integrative approach investigating the role of music beyond clinical applications on conceptual and emotional levels in persons with Parkinson's disease. Finally, important comprehensive reviews provide updates on the evidence base for music and NMT in upper extremity stroke rehabilitation (Ghai) and in cognitive interventions in Alzheimer's disease (Leggieri et al.).

The compilation of papers in this special Research Topic cannot claim complete representation of all important areas of translational research and effective clinical applications. For example, critical music based research areas in speech and language, hemi-spatial neglect, memory, executive, and psychosocial function are not covered but readers are encouraged to acquaint themselves with those results. They are fully covered in the NMT treatment model of 20 standardized clinical techniques across 3 rehabilitation domains. However, each of the papers in this topic presents critical new information in the continuous advancement of understanding the role and function of music as a complex auditory language in helping to advance brain rehabilitation. Twenty years ago, this topic would not have been possible as a major contribution to brain research and neurorehabilitation. The rehabilitative effects of music based interventions on meaningful clinical outcomes and brain plasticity were not known. However, the enormous progress in the basic neuroscience of music perception, cognition, and production has led to a translational clinical research path in music that has made the development and establishment of a medically recognized treatment system such as Neurologic Music Therapy possible in a comparatively short time span, from “bench” to “therapy.”

## Author Contributions

All authors listed have made a substantial, direct and intellectual contribution to the work, and approved it for publication.

## Conflict of Interest

VH was employed by company SRH Gesundheitszentrum Bad Wimpfen GmbH. The remaining authors declare that the research was conducted in the absence of any commercial or financial relationships that could be construed as a potential conflict of interest.

## Publisher's Note

All claims expressed in this article are solely those of the authors and do not necessarily represent those of their affiliated organizations, or those of the publisher, the editors and the reviewers. Any product that may be evaluated in this article, or claim that may be made by its manufacturer, is not guaranteed or endorsed by the publisher.

